# Classification of grape seed residues from distillation industries in Europe according to the polyphenol composition highlights the influence of variety, geographical origin and color

**DOI:** 10.1016/j.fochx.2024.101362

**Published:** 2024-04-07

**Authors:** Thibaut Munsch, Magdalena Anna Malinowska, Marianne Unlubayir, Manon Ferrier, Cécile Abdallah, Marin-Pierre Gémin, Kévin Billet, Arnaud Lanoue

**Affiliations:** a Université de Tours, EA 2106 « Biomolécules et Biotechnologies Végétales, UFR des Sciences Pharmaceutiques, 31 av. Monge, F37200 Tours, France; b Cracow University of Technology, Faculty of Chemical Engineering and Technology, 24 Warszawska St., 31-155 Cracow, Poland

**Keywords:** Grape seed residues, Polyphenols, Metabolomic, Multivariate statistical analysis

## Abstract

Grape seed residues represent the raw material to produce several value-added products including polyphenol-rich extracts with nutritional and health attributes. Although the impact of variety and environmental conditions on the polyphenol composition in fresh berries is recognized, no data are available regarding grape seed residues. The chemical composition of grape seed residues from wine distilleries in France, Spain and Italy was characterized by mass spectrometry. Forty-two metabolites were identified belonging to non-galloylated and galloylated procyanidins as well as amino acids. Polyphenol concentrations in the red varieties originated from Champagne or Veneto were twice higher than in white varieties from the Loire Valley. The chemical profiles of grape seed residues were mainly classified according to the color variety with galloylated procyanidins as biomarkers of white varieties and non-galloylated procyanidins as biomarkers of red ones. The present approach might assist the selection of grape seed residues as quality raw materials for the production of polyphenol-rich extracts.

## Highlights

• UPLC-MS-based metabolomic fingerprint was applied to various grape seed residues.
• 42 metabolites were identified mainly related to flavan-3-ols and procyanidins.
• Samples were discriminated according to color, variety and geographical origins.
• The red varieties of Pinot group contained highest polyphenol concentrations.
• Galloylated procyanidins were biomarkers of the white varieties.

## Graphical abstract

**Figure:**
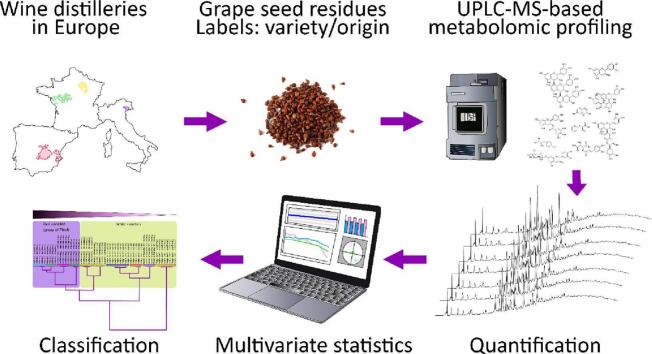

## 1 Introduction

Grape (*Vitis vinifera* L.) is one of the most valuated fruit crops on the world. In 2020, the global production of fresh grape reached 78 million tons with about 80% used for the winemaking industry (FAOSTAT, 2023). Grape pomace, the most abundant by-product of the wine industry, is produced after pressing and fermentation and consists of stalks, grape seeds and skins. In the western European countries, Spain, France and Italy, where the viticulture is an important agricultural activity, the production of grape pomace can reach 800,000 to 1,000,000 tons per year (OIV, 2018). The distilleries ensure the removal and the processing of grape pomace within a wine-producing region and a single company can process up to 90,000 t per year.

After distillation of grape pomace for alcohol production, the corresponding residues contain relevant concentrations of bioactive compounds notably condensed tannins, also called procyanidins (Devesa-Rey et al., 2011). Grape seed polyphenols have been reported for preventive and therapeutic use in Alzheimer's disease (Wang et al., 2009), chemoprevention of various cancers through antioxidant activities (Mancini et al., 2023) and the prevention of aortic atherosclerosis development in cardiovascular disease (Auger et al., 2004). Alternatively, grape seed-based bioactive compounds have been proposed for several industrial applications including cosmetics and nutraceutics (Salem et al., 2023).

The polyphenol fraction of grape seeds is composed by a complex mixture of monomeric flavan-3-ols as well as oligomeric and polymeric forms with high structural complexity (Ma et al., 2018; Pasini et al., 2019; Rockenbach et al., 2012). This peculiar chemical complexity makes it challenging to assess the quality and composition of grape seed residues used as raw material to produce commercial grape seed extracts with high procyanidin contents (Padilla-González et al., 2022).

Metabolomics aims to explore complex small-molecule profiles of a biological system from a given genotype under the influence of environmental factors (Fiehn, 2002). Metabolomics combined to chemometric methods was successfully applied to a variety of plant products to evaluate their quality, authenticity and safety and can also be used to address the geographical origin or the control of adulteration (Pereira et al., 2023; Sarkar et al., 2023). Grape metabolomics was relevant to classify different genotypes based on berries, wines and byproducts (Billet et al., 2018, Billet et al., 2021; Chira et al., 2009; Mattivi et al., 2006) and the signature of the geographical origin was revealed in wine and grape quality (Anesi et al., 2015; Canizo et al., 2018). Focusing on grape seed residues from ethanol-distillation industry, only few data are available. Different extracting methods have been proposed, showing the possible recovery of valuable polyphenols even after long thermal distillation (Peralbo-Molina et al., 2013) and HPLC methods were developed to control adulteration in grape seed extracts (Govindaraghavan, 2019; Villani et al., 2015). Nowadays, standardized extracts based on grape seed residues from selected varieties are released on the market, but no studies reported the influence of the variety and geographical origin. The development of analytical approaches is therefore required to assess the complex polyphenol composition in grape seed residues in order to assist the selection of raw materials.

During grapevine growth, the biosynthesis of flavan-3-ols starts before the flowering and increase until véraison with an accumulation in skin and seed of berries. During the berry development, the change in procyanidin composition is responsible for a seed color change from green to brown and this feature is used by winegrowers to estimate maturity stage. An increase of polymerization degree in grape seeds was observed during maturity but these observations remain controversial (Geny et al., 2003; Rousserie et al., 2019). Polyphenol metabolism plays a major role in plant adaptation to environmental stress including biotic and abiotic factors, consequently the occurrence of polyphenol variations in grape seed residues according to geographical origin may be suggested but was not reported. The impact of viticultural practices including leaf removal, water deficit irrigation or pruning was investigated as a possible cause of seed polyphenol changes, however no tangible impact was observed (Rousserie et al., 2019).

The aim of the study was to assess the variability of polyphenol composition in grape seed residues from several distilleries in Europe covering 8 grape varieties and 4 wine-producing regions in Europe. UPLC-MS-based semi-targeted metabolomic profiling was applied to identify the compounds from the extracts of grape seed residues. The major polyphenols were quantified and allowed a ranking of raw materials according to polyphenol contents. Chemometric tools including principal component analysis (PCA), hierarchical cluster analysis (HCA) and orthogonal partial least squares discriminant analysis (OPLS-DA) were used to classify the samples and propose biomarkers.

## 2 Materials and methods

### 2.1 Chemicals

LC-MS grade acetonitrile and formic acid, analytical grade methanol and chloroform were all purchased from Fisher Scientific (Illkirch-Graffenstaden, France). Ultrapure water was purified from a Milli-Q water system (Merck Millipore, Molsheim, France). Pure standards of catechin, epicatechin, *L*-phenylalanine, citric acid, L-tryptophan, L-tyrosine, L-isoleucine, L-leucine and gallic acid were purchased from Sigma-Aldrich (St. Louis, MO, USA). Procyanidins B1, B2, B3 and C1 were supplied by Extrasynthese (Genay, France), procyanidin B4 and caftaric acid were obtained from Carbosynth (Compton, Berkshire, UK).

### 2.2 Plant material

**Fig. 1:**
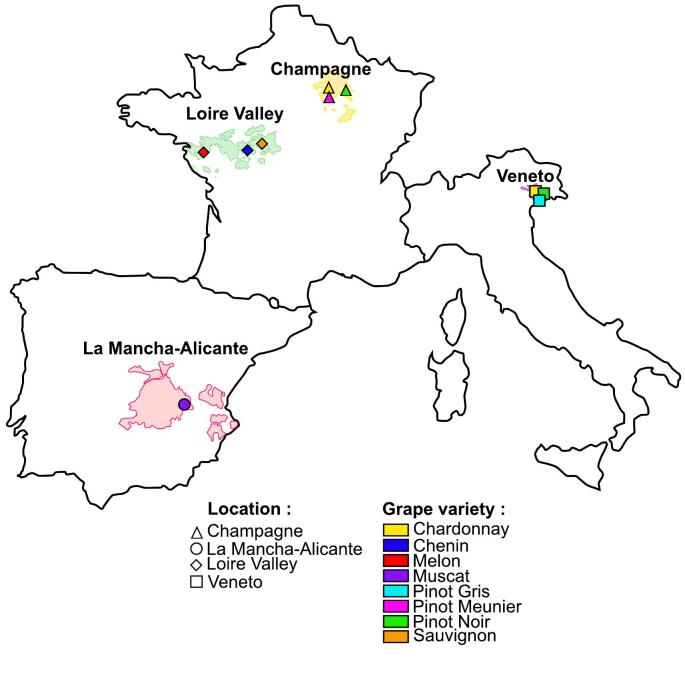
Location of wine-producing areas were grape seed residues of different varieties were collected for the present study.

Grape (*Vitis vinifera* L.) seeds were collected in 2018 as by-products of four distilleries located in different wine-producing areas in Europe: Distillerie du Vouvray and Distillerie Baron (Loire Valley; France), Distillerie Jean Goyard (Champagne; France), Distillerie Bonollo (Veneto; Italy) and La Mancha-Alicante (Alvinesa; Spain) (Fig. 1). The collected varieties corresponded to the dominant varieties present on the wine-producing areas: ie. Chardonnay, Pinot Meunier, Pinot Noir (Champagne, France); Chenin, Melon, Sauvignon (Loire Valley, France); Chardonnay, Pinot Noir, Pinot Gris (Veneto, Italy); and Muscat from La Mancha-Alicante (Spain). Finally, combining varietal and geographical origins, a total of 10 different grape seeds were collected.

### 2.3 Samples preparation

Polyphenol extraction from grape seed residues was based on (Narduzzi et al., 2015). Grape seed samples (10 × 5 replicates) were ground for 2 min in a cooled analytical mill (Ika-Werke A10, Staufen, Germany). Fifty mg of each sample powder were extracted with 1 mL of methanol/water/chloroform (2:1:2; *v*/*v*/v) mixture containing 0.1% formic acid. The samples were then placed for 1 h in an ultrasonic bath filled with ice (AL04–12-230, Advantage lab) and centrifuged for 10 min at 16,800*g* at 4 °C. The upper aqueous phase (400 μL) was collected and added to 600 μL of water/acetonitrile (95:5, v/v) acidified with 0.1% formic acid. The samples were centrifuged a second time for 10 min at 18000 rpm at 4 °C. The supernatants were stored at −20 °C prior to UPLC-DAD-MS analyses.

### 2.4 UPLC-DAD-MS analyses

Semi-targeted UPLC-DAD-MS method was adapted from a previous study (Billet et al., 2020) using a Xevo TQD mass spectrometer operated in positive and negative ionization modes (Waters, Milford, MA). Analytes were eluted with a linear gradient from 5 to 30% of solvent B (acetonitrile containing 0.1% formic acid) using an ACQUITY UPLC HSS T3 1.8 μm (2.1 × 150 mm) column (Waters, Milford, MA). The solvent A consisted in water containing 0.1% formic acid. Quality control (QC) samples represented the mixture of all samples from the study and were regularly injected every 10 samples during the batch.

### 2.5 Treatment of MS data

Full scan data acquisition modes in the range 50–2000 *m/z* were used for the metabolic profiling of grape seed extracts from the 10 different origins. Analyte identification was established following retention times, *m/z* values and UV spectra by comparison with commercial standards, or data from the literature when no standards were available. Moreover, electrospray ionization (ESI) in-source fragmentation provided key information for the identification. Once metabolic profiling was completed, quantitative UPLC-MS analyses were performed using selected ion monitoring (SIM) mode by targeting the 42 molecular ions, in either [M + H]^+^ or [M-H]^−^. The generated chromatograms were integrated using the application TargetLynx of MassLynx 4.2 software. Every integrated peak was visually checked and manually corrected if necessary. Absolute quantification was performed for catechin, epicatechin, procyanidins B1-B4 and procyanidin C1 using 6-points calibration curve (0–10 ppm) of pure standards. Standards were injected in the same analytical conditions and in the sample set as grape seed samples. Quantification was achieved through selected ion monitoring (SIM) mode as described above, targeting the *m/z* corresponding to [M-H]^−^ ions.

### 2.6 Statistical analyses

Multivariate statistical analysis were conducted on SIMCA 17.0 (Umetrics AB, Umeå, Sweden) software. Principal Component Analysis (PCA) was applied for all the samples and Hierarchical Cluster Analysis (HCA) was performed using Ward's method. Co-occurrence networks were established as previously described (Billet et al., 2023). Orthogonal Partial Least Squares Discriminate Analysis (OPLS-DA) was conducted according to the color variety to identify the Variable Important in Projection (VIP > 1). Kruskal-Wallis's tests were used for non-parametric univariate statistics.

## 3 Results and discussion

### 3.1 Metabolomics profiling of grape seed residues

Semi-targeted metabolomics method was developed specifically on the extracts of grape seed residues by using positive- (ESI^+^) and negative-ion (ESI^−^) electrospray ionization in full-scan mode, resulting in the identification of 42 analytes (Table 1). For 17 analytes, the putative molecular assignments were authenticated by comparison with pure standards (confidence level 1 in metabolite identification)(Sumner et al., 2007) corresponding to citric acid (m1), L-tyrosine (m2), L-isoleucine (m3), L-leucine (m4), gallic acid (m5), *L*-phenylalanine (m6), protocatechuic acid (m9), caftaric acid (m10), L-tryptophan (m12), procyanidin B1 (m14), coutaric acid (m15), procyanidin B3 (m16), catechin (m17), procyanidin B4 (m21), procyanidin B2 (m23), epicatechin (m27) and procyanidin C1 (m30). Other analytes were putatively identified (level 2) and were the following: Peaks m7, m13, m18, m20, m22, m25 and m33, showed similar patterns of ionization compared to procyanidin C1 (m30), with an [M-H]^−^ ion at *m/z* 865 giving a fragment at *m/z* 577 (loss of catechin unit) and a [M + H]^+^ at *m/z* 867 giving a fragment at *m/z* 579 (loss of catechin unit). Consequently, these 7 analytes were temporarily assigned to C-type (trimeric) procyanidins annotated with letters ranged from a to g. Peak m8 showed a molecular ion [M-H]^−^ at *m/z* 331 and [M + H]^+^ at *m/z* 333, producing fragments at *m/z* 169 (ES^−^) and 171 (ES^+^) corresponding to gallic acid moiety (−162 Da, glucose loss). Therefore, this compound was identified as monogalloyl glucose. Peak m11 with molecular ions [M-H]^−^ at *m/z* 451 and [M + H]^+^ at *m/z* 453 showed fragments at *m/z* 289 (ES^−^) and 291 (ES^+^) (−162 Da, glucose loss) corresponding to catechin and was temporarily assigned to catechin glucoside (Narduzzi et al., 2015). Peaks m19, m26, m29, m34 showed identical molecular ions; [M-H]^−^ at *m/z* 1153, [M-2H]^2−^ at *m/z* 576, [M + H]^+^ at *m/z* 1155, [M + 2H]^2+^ at *m/z* 578 and [M-H-catechin]^−^ ion at *m/z* 865. Therefore, these group of compounds were tentatively identified as D-type (tetrameric) procyanidins (Narduzzi et al., 2015).

**Table 1:** List of compounds identified in the studied grape seed extracts.

Peak	RT (min)	Compound class	Compound assignment	Molecular ion adducts ES^+^	In-source fragment ES^+^	Molecular ion adducts ES^−^	In-source fragment ES^−^	ʎ_max_ (nm)	References
m1	1.42	organic acid	citric acid			191 [M-H]^−^	111		standard
m2	1.43	amino acid	L-tyrosine	182 [M + H]^+^	165				standard
m3	1.64	amino acid	L-leucine	132 [M + H]^+^	86				standard
m4	1.78	amino acid	L-isoleucine	132 [M + H]^+^	86			215, 266	standard
m5	2.00	phenolic acid	gallic acid	171 [M + H]^+^	153 [M + H-H_2_O]^+^	169 [M-H]^−^	125	215, 269	standard
m6	2.80	amino acid	*L*-phenylalanine	166 [M + H]^+^	120	164 [M-H]^−^			standard
m7	2.95	procyanidin C	procyanidin C a	867 [M + H]^+^	579	865 [M-H]^−^	577, 423	278	(Narduzzi et al., 2015) (Simirgiotis & Schmeda-Hirschmann, 2010)
m8	3.15	phenolic acid	monogalloyl glucose	333 [M + H]^+^	171 [M + H-glucose]^+^	331 [M-H]^−^ 663 [2 M-H]^−^	169 [M-H-glucose]^−^ 125	210, 283	(Chedea et al., 2011)
m9	3.62	phenolic acid	protocatechuic acid	155 [M + H]^+^		153 [M-H]^−^	109	202, 218, 259, 292	standard
m10	4.18	phenolic acid	caftaric acid			311 [M-H]^−^	179, 149	221, 247, 297, 327	standard
m11	4.24	flavan-3-ol	catechin glucoside	453 [M + H]^+^	291 [M + H-glucose]^+^	451 [M-H]^−^ 903 [2 M-H]^−^	289 [M-H-glucose]^−^	203, 277	(Narduzzi et al., 2015)
m12	4.44	amino acid	L-tryptophan	205 [M + H]^+^	188	203 [M-H]^−^		218, 278	standard
m13	5.08	procyanidin C	procyanidin C b	867 [M + H]^+^	579	865 [M-H]^−^	577, 451, 289		(Narduzzi et al., 2015) (Simirgiotis & Schmeda-Hirschmann, 2010)
m14	5.30	procyanidin B	procyanidin B1	579 [M + H]^+^	427, 409, 291	577 [M-H]^−^ 1155 [2 M-H]^−^	425, 289	278	standard
m15	5.61	phenolic acid	coutaric acid			295 [M-H]^−^	163		standard
m16	5.65	procyanidin B	procyanidin B3	579 [M + H]^+^ 1157 [2 M + H]^+^	427, 289	577 [M-H]^−^ 1155 [2 M-H]^−^	425, 289	278	standard
m17	5.89	flavan-3-ol	catechin	291 [M + H]^+^	139, 123	289 [M-H]^−^ 579 [2 M-H]^−^	245	225, 278	standard
m18	6.22	procyanidin C	procyanidin C c	867 [M + H]^+^	579, 289	865 [M-H]^−^ 1731 [2 M-H]^−^	577, 434	278	(Narduzzi et al., 2015) (Simirgiotis & Schmeda-Hirschmann, 2010)
m19	6.36	procyanidin D	procyanidin D a	1155 [M + H]^+^ 578 [M + 2H]^2+^		1153 [M-H]^−^ 576 [M-2H]^2−^	865 [M-H-catechin]^−^, 293	278	(Narduzzi et al., 2015)
m20	6.49	procyanidin C	procyanidin C d	867 [M + H]^+^	579	865 [M-H]^−^	577, 451, 289	278	(Narduzzi et al., 2015) (Simirgiotis & Schmeda-Hirschmann, 2010)
m21	6.63	procyanidin B	procyanidin B4	579 [M + H]^+^	409, 289	577 [M-H]^−^ 1155 [2 M-H]^−^	425, 289	278	standard
m22	6.79	procyanidin C	procyanidin C e	867 [M + H]^+^	579	865 [M-H]^−^	577, 289	218, 278	(Narduzzi et al., 2015) (Simirgiotis & Schmeda-Hirschmann, 2010)
m23	6.96	procyanidin B	procyanidin B2	579 [M + H]^+^	427, 4097, 289	577 [M-H]^−^ 1155 [2 M-H]^−^	425, 289	278	standard
m24	7.08	galloyl procyanidin C	galloyl procyanidin C a	1019 [M + H]^+^		1017 [M-H]^−^ 508 [M-2H]^2−^	864 [M-H-galloyl]^−^ 720	217, 278	(Narduzzi et al., 2015)
m25	7.39	procyanidin C	procyanidin C f	867 [M + H]^+^	579, 291	865 [M-H]^−^	577, 451, 289	278	(Narduzzi et al., 2015)
m26	7.55	procyanidin D	procyanidin D b	1155 [M + H]^+^ 578 [M + 2H]^2+^	441, 285	1153 [M-H]^−^ 576 [M-2H]^2−^	1017, 865 [M-H-catechin]^−^	278	(Narduzzi et al., 2015)
m27	7.80	flavan-3-ol	epicatechin	291 [M + H]^+^	139, 123	289 [M-H]^−^ 579 [2 M-H]^−^	245	224, 278	standard
m28	8.03	galloyl procyanidin C	galloyl procyanidin C b	1019 [M + H]^+^		1017 [M-H]^−^ 508 [M-2H]^2−^	864 [M-H-galloyl]^−^ 729	276	(Narduzzi et al., 2015)
m29	8.38	procyanidin D	procyanidin D c	1155 [M + H]^+^ 578 [M + 2H]^2+^	289.0	1153 [M-H]^−^ 576 [M-2H]^2−^	865 [M-H-catechin]^−^, 432	278	(Narduzzi et al., 2015)
m30	8.57	procyanidin C	procyanidin C1	867 [M + H]^+^	579, 289	865 [M-H]^−^ 1731 [2 M-H]^−^	577, 451, 289	278	standard
m31	8.71	galloyl procyanidin B	galloyl procyanidin B a	731 [M + H]^+^	579 [M + H-galloyl]^+^	729 [M-H]^−^	577 [M-H-galloyl]^−^	278	(Narduzzi et al., 2015)
m32	8.99	galloyl procyanidin B	galloyl procyanidin B b	731 [M + H]^+^	579 [M + H-galloyl]^+^	729 [M-H]^−^ 366 [M-2H]^2−^	577 [M-H-galloyl]^−^	220, 278	(Narduzzi et al., 2015)
m33	9.81	procyanidin C	procyanidin C g	867 [M + H]^+^	579, 270.9	865 [M-H]^−^ 1731 [2 M-H]^−^	577	216, 278	(Narduzzi et al., 2015)
m34	10.27	procyanidin D	procyanidin D d	1155 [M + H]^+^ 578 [M + 2H]^2+^		1153 [M-H]^−^ 576 [M-2H]^2−^	865 [M-H-catechin]^−^, 289	278	(Narduzzi et al., 2015)
m35	10.83	galloyl-flavan-3-ol	catechin/epicatechin gallate	443 [M + H]^+^	291 [M + H-galloyl]^+^	441 [M-H]^−^ 883 [2 M-H]^−^	289 [M-H-galloyl]^−^ 169 [galloyl-H]^−^	276	(Narduzzi et al., 2015)
m36	11.18	galloyl procyanidin C	galloyl procyanidin C c	1019 [M + H]^+^		1017 [M-H]^−^ 508 [M-2H]^2−^	796	278	(Narduzzi et al., 2015)
m37	11.37	galloyl procyanidin D	galloyl procyanidin D	1308 [M + H]^+^	579	1306 [M-H]^−^ 652 [M-2H]^2−^	577	278	(Spencer et al., 2007)
m38	12.80	galloyl procyanidin C	galloyl procyanidin C d	1019 [M + H]^+^		1017 [M-H]^−^	865 [M-H-galloyl]^−^ 577	278	(Narduzzi et al., 2015)
m39	12.90	galloyl procyanidin C	galloyl procyanidin C e	1019 [M + H]^+^		1017 [M-H]^−^ 508 [M-2H]^2−^	577	216, 266, 353	(Narduzzi et al., 2015)
m40	13.01	galloyl-flavan-3-ol	galloyl catechin A a	441 [M + H]^+^	289 [M + H-galloyl]^+^	439 [M-H]^−^	287 [M-H-galloyl]^−^	231, 266, 296, 350	(Narduzzi et al., 2015)
m41	13.68	gall-procyanidin B	galloyl procyanidin B c	731 [M + H]^+^	577 [M + H-galloyl]^+^	729 [M-H]^−^	577 [M-H-galloyl]^−^	278	(Narduzzi et al., 2015)
m42	13.87	galloyl-flavan-3-ol	galloyl catechin A b	441 [M + H]^+^	289 [M + H-galloyl]^+^	439 [M-H]^−^	287 [M-H-galloyl]^−^	229, 267, 298, 345	(Narduzzi et al., 2015)

Another group of compounds, peaks m24, m28, m36, m38 and m39, showed identical patterns of ionization with [M-H]^−^ ion at *m/z* 1017, [M-2H]^2−^ at *m/z* 508, [M + H]^+^ at *m/z* 1019 producing a fragment [M-H-galloyl]^−^ ion at *m/z* 864. Therefore, these compounds were annotated as C-type (trimeric) galloyl-procyanidins (Narduzzi et al., 2015). Peaks m31, m32 and m41 showed [M-H]^−^ ion at *m/z* 729, [M + H]^+^ ion at *m/z* 731 producing a daughter ion [M-H-galloyl]^−^ at *m/z* 577 and were assigned to B-type (dimeric) galloyl-procyanidins (Narduzzi et al., 2015). Peak m37 presented [M-H]^−^ ion at *m/z* 1306, [M-2H]^2−^ ion at *m/z* 652 as well as [M + H]^+^ ion at *m/z* 1308. Therefore, this compound was temporarily assigned to D-type (tetrameric) galloyl-procyanidin (Spencer et al., 2007). Peaks m40 and m42 showed identical patterns of ionization with [M-H]^−^ at *m/z* 439, [M + H]^+^ ion at *m/z* 441 producing the fragments [M + H-galloyl]^+^ at *m/z* 289 and [M-H-galloyl]^−^ at *m/z* 287 and were assigned to galloylcatechin A (Narduzzi et al., 2015).

**Fig. 2:**
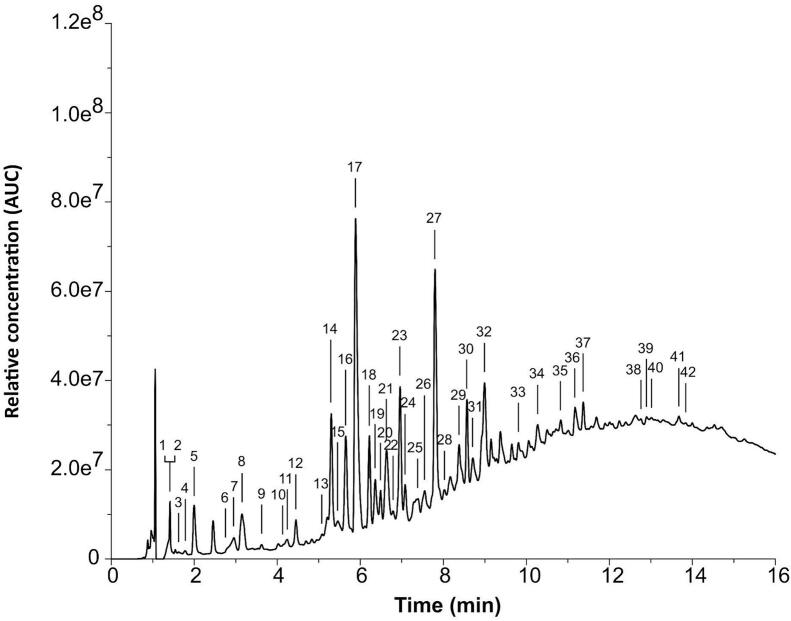
UPLC-DAD-MS chromatographic profile of grape seed extract from a quality control sample. The identification of the annotated peaks is presented in Table 1.

UPLC-DAD-MS chromatograms (Fig. 2) presented a typical baseline increase from 5 min, called “unsolved hump” or “bulge”, explained by the elution of condensed tannins with high degrees of polymerization (until DP20) (Villani et al., 2015; Ma et al., 2018; Peng et al., 2001, Rockenbach et al., 2012, Tsang et al., 2005;). Considering the glycosylated metabolites, only two compound; catechin-glucoside and monogalloyl glucose could be detected in the present study. It is much less compared to the 14 different flavan-3-ol monoglycosides reported for fresh grape seeds (Delcambre & Saucier, 2012). The apparent loss of glycosylated compounds in grape seeds as distillery by-products compared to grape seeds from fresh berries could be explained by a thermal deglycosylation occuring during the distillation of grape pomace.

### 3.2 Absolute quantification

**Fig. 3:**
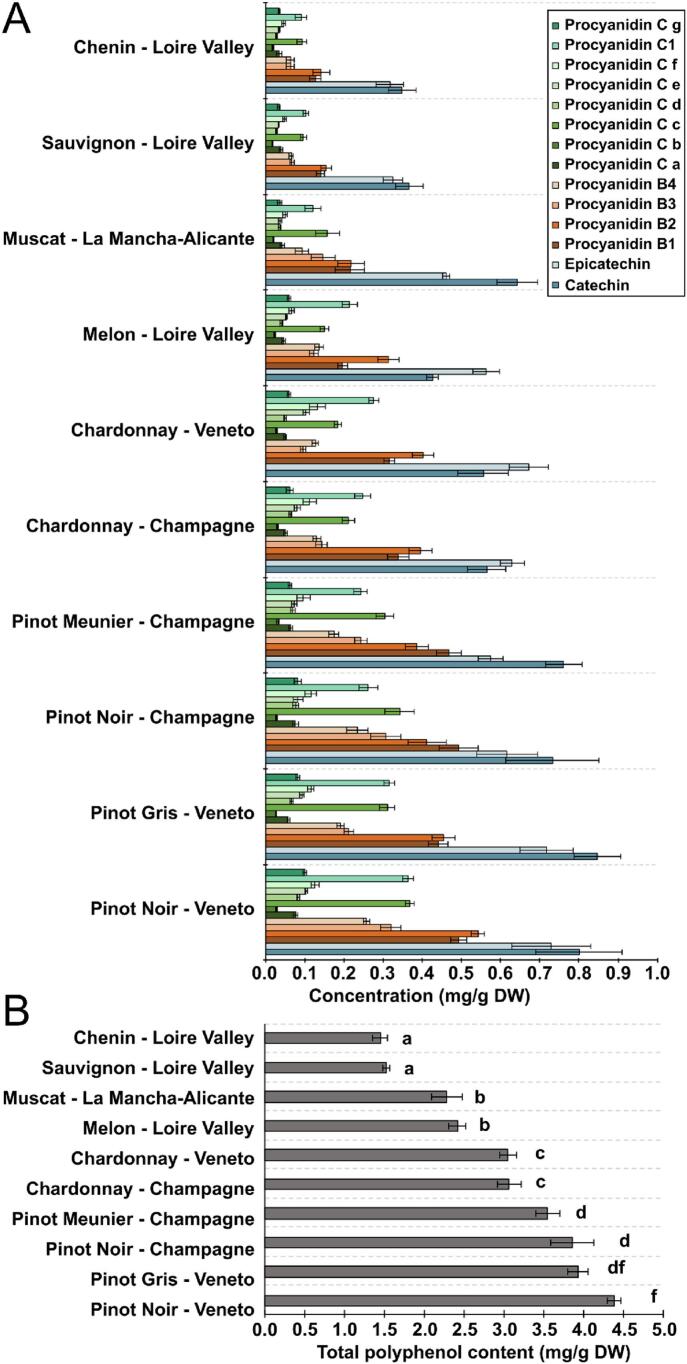
Concentration of single polyphenols (A) total polyphenol concentration (B) in extracts from grape seed residues. Error bars represent the standard deviation. Significant differences were found between values with different letters (ANOVA, *p*-value <0.05).

The absolute quantification of the major polyphenols (catechin, epicatechin, procyanidins B1–4, procyanidin C1 and C-type procyanidins) of grape seed extracts from five white grape cultivars (Chardonnay, Chenin, Melon, Muscat, Sauvignon) and three red grape cultivars (Pinot Gris, Pinot Meunier, Pinot Noir) was performed by UPLC-MS and SIM mode (Fig. 3A; Table S1). The monomeric flavan-3-ols (catechin and epicatechin) were the two major compounds in all tested samples, with highest concentrations observed in the red grape varieties compare to white ones. The highest concentration of catechin was detected in Pinot Gris and Pinot Noir from Veneto with 0.85 ± 0.06 and 0.80 ± 0.10 mg/g DW respectively, as well as in Pinot Meunier and Pinot Noir from Champagne with 0.76 ± 0.04 and 0.73 ± 0.11 mg/g DW, respectively. White cultivars were characterized by relatively lower concentrations of monomeric flavan-3-ols. The lowest concentrations in catechin were observed in Sauvignon and Chenin from Loire Valley with 0.36 ± 0.03 and 0.34 ± 0.03 mg/g DW, respectively. Following the monomeric flavan-3-ols, several B-type (dimeric) procyanidins were the most abundant compounds, with highest concentrations observed in the red grape varieties compare to the white ones. Among procyanidins B1, B2, B3 and B4, the concentrations of B1 and B2 were often higher than B3 and B4. The concentrations of procyanidin B1 were maximal in Pinot Noir, Pinot Gris and Pinot Meunier from Champagne and Veneto with concentrations ranged from 0.43 ± 0.03 to 0.49 ± 0.02 mg/g DW, whereas it was only 0.12 ± 0.01 and 0.14 ± 0.01 mg/g DW in Chenin and Sauvignon from Loire Valley, respectively. The levels in procyanidin B2 were maximal in Pinot Noir, Pinot Gris and Pinot Meunier from Champagne and Veneto with concentrations ranged from 0.38 ± 0.02 to 0.54 ± 0.01 mg/g DW, whereas Chenin from Loire Valley contained only 0.14 ± 0.02 mg/g DW. Procyanidin B3 was accumulated in the range of 0.06 ± 0.01 (Sauvignon from Loire Valley) to 0.31 ± 0.02 mg/g DW (Pinot Noir from Veneto) and Procyanidin B4 from 0.06 ± 0.01 (Sauvignon from Loire Valley) to 0.25 ± 0.01 mg/g DW (Pinot Noir from Veneto). The levels in procyanidin C1 were maximal in Pinot Noir, Pinot Gris and Pinot Meunier from Champagne and Veneto with concentrations ranged from 0.24 ± 0.01 to 0.36 ± 0.01 mg/g DW, whereas Chenin from Loire Valley contained only 0.01 ± 0.001 mg/g DW. In all varieties procyanidin C1 concentration was higher than others C-type procyanidins (Fig. 3A; Table S1).

Fig. 3B presents the ranking of the polyphenol contents in grape seed residues as the sum of catechin, epicatechin, procyanidins B1–4, procyanidin C1 and C-type procyanidins. Pinot Noir, Pinot Gris and Pinot Meunier from Champagne and Veneto showed the highest concentrations with >4.39 ± 0.51 mg/g DW, whereas Chenin and Sauvignon accumulated <1.44 ± 0.17 mg/g DW (Table S1). Whereas procyanidins and flavan-3-ols were usually analyzed in grape seeds of fresh berries or during winemaking process, no report examined the correspond levels in grape seed as by-products of distilleries. The present catechin and epicatechin levels for grape seed residues were much lower compared to those described in grape seeds of fresh berries (Bozan et al., 2008; Chira et al., 2009; Popov et al., 2017; Yilmaz & Toledo, 2004). Indeed, a great portion of seed polyphenols are extracted during winemaking process and latter during pomace distillation a thermal degradation is likely to occur (Cisneros-Yupanqui et al., 2022; Rousserie et al., 2019). Interestingly, the present ranking of cultivars according to polyphenol contents corresponded to previous results in grape seeds of fresh berries with high accumulations in Pinot Noir and Pinot Gris and low ones in Chardonnay, Muscat and Sauvignon (Popov et al., 2017).

### 3.3 Multivariate statistical analyzes

**Fig. 4:**
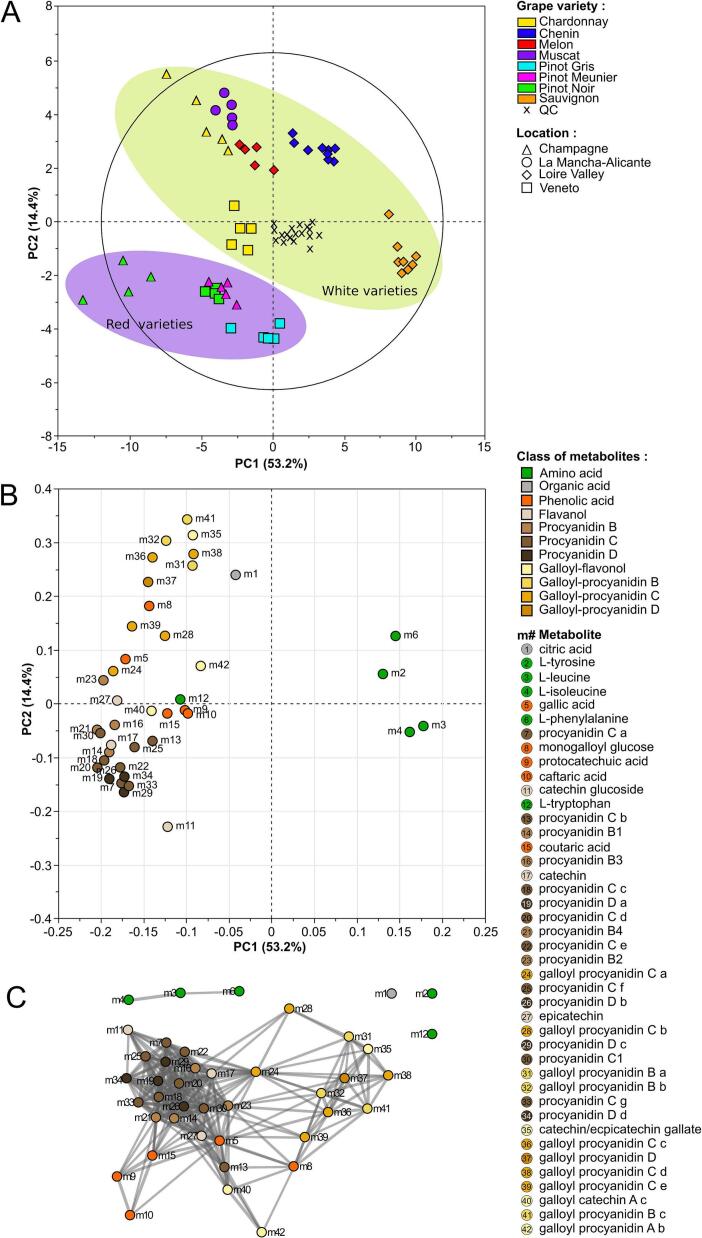
Unsupervised classification using principal component analysis on metabolomic data extracts of grape seed residues. In score plot (A) colors correspond to varieties and symbols to geographical origins. In loading plot (B) colors correspond to the metabolic class and numbers to metabolite name. Co-occurrence networks on metabolites from extracts of grape seed residues (C). Threshold: *R* > 0.6 and *p*-value <0.05. Short node distance indicates high correlation.

PCA was performed to show similarities and differences in the metabolomic composition of grape seed extracts depending on varietal and geographical origin (Fig. 4). The PCA score plot explained 67.6% of the dataset variability on the two first principal components, with the first principal component (PC1) accounting for 53.2% and the second (PC2) for 14.4% of the overall variance. Quality control samples (QC) appeared well grouped at the intersection of PC1 and PC2 ensuring the robustness of the measurements and the low analytical variability. A perfect separation of sample groups was obtained according to the grape varietal color as represented by the two ellipses on PCA score plot (Fig. 4A). Additionally, most of the sample groups appeared well grouped, highlighting specific metabolomic compositions (metabotypes) according to varietal and geographical origins of grape seeds. Two sample groups showed overlapping (Pinot Meunier from Champagne and Pinot Noir from Veneto), thereby revealing a close phytochemical composition. Interestingly, a supergroup of samples was projected on PC1 and PC2 negative scores corresponding to Pinot Noir, Pinot Gris and Pinot Meunier. These group of metabotypes presented some similarities even when they originated from two geographical origins (Pinot Noir from Veneto and Champagne) and corresponded to closely related genotypes previously called “Noiriens” when defined as eco-geographical group by ampelographers (Bisson, 1999; Levadoux, 1948). Nevertheless, the PCA score plot also enables to assess the impact of geographical origin, as it is shown by the separation of the two sample groups of Chardonnay and Pinot Noir originated either from Champagne or Veneto.

The loading plot (Fig. 4B) presented the underlying metabolites responsible for the separations, with polyphenol-rich metabotypes that were projected on PC1 negative in opposite direction to metabotypes showing over accumulation of amino acids in PC1 positive except for L-tryptophan (m12). As an example, Sauvignon samples projected in PC1 positive were described by relative high accumulations in L-tyrosine (m2), L-leucine (m3), L-isoleucine (m4) and *L*-phenylalanine (m6) and poor levels in procyanidins. However, Pinot Noir and Chardonnay originated from Champagne, projected in PC1 negative, presented high content in all procyanidins and few amounts in amino acids. These opposition could be explained by the trade-off between primary and secondary metabolism in plants (Neilson et al., 2013). Variations along PC2 axis corresponded to relative compositions of galloylated and non-galloylated procyanidins. Red grape varieties, projected on PC2 negative, presented higher content of dimeric (m14, m16, m21, m23), trimeric (m7, m13, m18, m20, m22, m25, m30, m33) and tetrameric (m19, m26, m29, m34) procyanidins. While white grape varieties, projected on PC2 positive, presented higher levels of galloylated procyanidins (dimers: m31, m32, m41; trimers: m24, m28, m36, m38, m9; tetramers: m37). Consequently, galloylated procyanidins appeared as biomarkers of white grape varieties in grape seeds, and non-galloylated procyanidins as biomarkers of red grape varieties. The presence of galloyl groups is known to affect the physicochemical properties of polyphenols and usually galloylation improves biological activities of procyanidins by increasing their bioavailability (Karas et al., 2017). In the future, it could be therefore interesting to develop grape seed extracts based on selected white varieties rich in galloylated procyanidins with the aim to improve the bioavailability of bioactive compounds. A co-occurrence network based on the 42 metabolites was performed to reveal similar patterns of accumulation among the 8 tested genotypes from 4 geographical origins (Fig. 4C). It showed 507 significant positive correlations at threshold: *R* > 0.6 and *p-*value <0.05. Short node distance (Pearson correlation coefficients) indicates high correlation. As a result, the correlation network showed that structurally related compounds were intercorrelated and clustered together. Polyphenols were highly correlated in a supercluster showing specific subclusters depending on the degree of oligomerization and galloylation of procyanidins. Three amino acids; L-isoleucine (m3), L-leucine (m4) and *L*-phenylalanine (m6) were also correlated in a second cluster.

### 3.4 Hierarchical clustering analysis

**Fig. 5:**
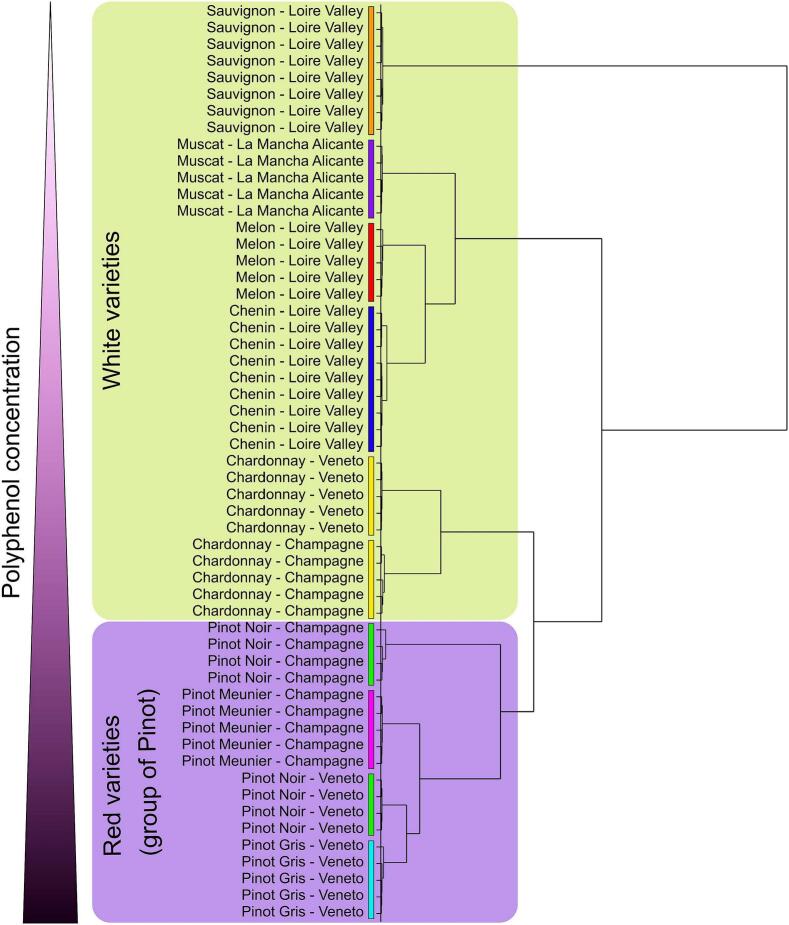
Dendrogram of hierarchical cluster analyses based on Ward's clustering of metabolomic data.

To go further in the classification of grape seed residues, HCA was applied on the loading matrix based on the relative abundance of the 42 metabolites. The dendrogram showed overall structural similarities of metabotypes determined by Ward's clustering based on Euclidean distance (Fig. 5). A perfect separation of all sample groups was observed on the HCA, thus enabling the discrimination of grape seed residue by varietal and geographical origins. Interestingly, sample positions in the dendrogram correspond to the order observed when ranked by total polyphenol content (Fig. 3). The dendrogram structure showed subgroups, suggesting different degree of similarities between metabotypes. A subgroup was constituted of the three red varieties, namely Pinot Noir, Pinot Gris and Pinot Meunier, corresponding also to the “Noiriens” group.(Bisson, 1999; Levadoux, 1948) On the other hand, the five white varieties, Sauvignon, Muscat, Melon, Chenin and Chardonnay, were grouped together. We observed that sample groups corresponding to Chardonnay from Veneto and Champagne were positioned close to the “Noiriens” group. Previous attempts of classification based on metabolomics analyses of grape cane extracts reported close similarities between the metabotypes of Chardonnay and Pinot Noir (Billet et al., 2018). The direct lineage of Chardonnay from Pinot Noir, as confirmed by genetic studies (Lacombe et al., 2013), could explained the closeness of these metabotypes. Sauvignon from Loire Valley presented the furthest chemical signature from other sample groups explained by high amino acid amounts and low polyphenol contents.

### 3.5 Variable selection for the identification of color biomarkers

**Fig. 6:**
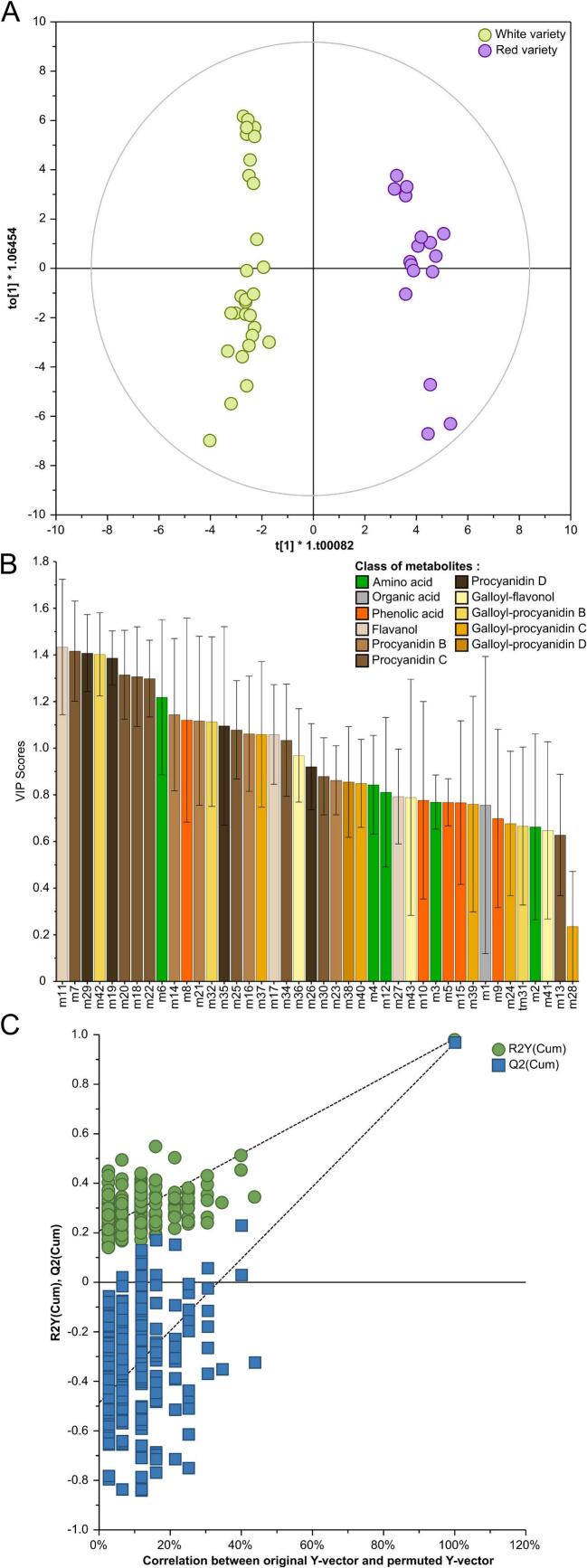
Supervised classification using OPLS-DA with “color variety” as discriminant variable on metabolomic data of grape seed extracts (A). VIP-scores of the OPLS-DA (B). The color corresponds to the polyphenol class and the numbers to the metabolite name (Fig. 4). Validation plot of 200 permutation tests for OPLS-DA model built for grape seed extracts.

Orthogonal partial least squares discriminant analysis (OPLS-DA) was performed as supervised multivariate analysis with “color variety” as discriminant variable (Fig. 6A). OPLS-DA model showed two groups well separated along the first component (model diagnostic: R^2^Xcum = 65.4%, R^2^Ycum = 97.6% and Q^2^cum = 96.9%). Biomarkers of color were established by multivariate (VIP > 1) and univariate statistics (*p* < 0.05; Kruskal-Wallis test). The VIP method (Fig. 6B) allowed the selection of 19 color biomarkers belonging to flavan-3-ols (m11, m17), procyanidins B (m14, m16, m21), procyanidins C (m7, m18, m20, m22, m25, m34), procyanidins D (m19, m29, m35), galloyl procyanidin C (m37), galloyl procyanidin B (m32, m42), phenolic acid (m8) and amino acid (m6). The statistical difference of the 19 biomarkers was controlled by one-way ANOVA with Kruskall-Wallis's test (p < 0.05). Additionally, permutation test (200 times) confirmed the ability to discriminate the grape seed extracts according to the color variety (Fig. 6C). Indeed, Y-axis intercepts R^2^ and Q^2^ were < 0.3 and < 0.05, respectively, and *p*-value of CV-ANOVA was <0.05.(Eriksson et al., 2013) The predictive model was confirmed by AUC (area under the curve) from the ROC curve (=1) and the correct classification rate (CCR; 100%).

## 4 Conclusions

Semi-targeted metabolomic approach was applied to characterize the composition of grape seed residues from several distilleries in Europe covering 8 grape varieties and 4 wine-producing regions in Europe. Forty-two metabolites were identified belonging to non-galloylated and galloylated procyanidins (dimers, trimers and tetramers) as well as amino acids. Polyphenol concentrations as the sum of catechin, epicatechin, procyanidins B1–4, procyanidin C1 and C-type procyanidins, were twice higher in red varieties compared to white varieties, however galloylated procyanidins were higher in white varieties and represented biomarkers of these white varieties. This work and its findings based on metabolic profiling coupled to multivariate statistical analyses might assist the selection of grape seed residues as quality raw materials for the production of polyphenol-rich grape seed extracts. The choice of the variety could be guided by the extent of oligomerization and galloylation exhibited by procyanidins and the sought biological activities.

The following is the supplementary data related to this article.

Table S1 Polyphenol concentrations (mg/g DW) in grape seed residues determined by UPLC-DAD-MS. Significant differences were found between values with different letters (ANOVA, *p*-value <0.05). Table S1

## CRediT authorship contribution statement

**Thibaut Munsch:** Writing – original draft, Methodology, Investigation. **Magdalena Anna Malinowska:** Writing – original draft, Conceptualization. **Marianne Unlubayir:** Methodology, Investigation. **Manon Ferrier:** Investigation, Data curation, Methodology. **Cécile Abdallah:** Data curation, Formal analysis, Visualization. **Marin-Pierre Gémin:** Data curation, Formal analysis, Visualization. **Kévin Billet:** Visualization, Validation, Software, Formal analysis, Data curation. **Arnaud Lanoue:** Writing – review & editing, Supervision, Project administration, Funding acquisition.

## Declaration of competing interest

The authors declare that they have no known competing financial interests or personal relationships that could have appeared to influence the work reported in this paper.

## Data Availability

Data will be made available on request.
