# Supplementary material for: Classification of grape seed residues from distillation industries in Europe according to the polyphenol composition highlights the influence of variety, geographical origin and color
Source: Food Chem X. 2024 Apr 7;22:101362. doi: 10.1016/j.fochx.2024.101362 (PMC11021364; doi:10.1016/j.fochx.2024.101362)
Supplement: Table S1 — Polyphenol concentrations (mg/g DW) in grape seed residues determined by UPLC-DAD-MS. Significant differences were found between values with different letters (ANOVA, p-value <0.05). [file mmc1.docx]

**Table S1.** Polyphenol concentrations (mg/g DW) in grape seed residues determined by UPLC-DAD-MS. Significant differences were found between values with different letters (ANOVA, *p-*value < 0.05).

|  | Pinot Noir - Veneto | Pinot Gris - Veneto | Pinot Noir - Champagne | Pinot Meunier - Champagne | Chardonnay - Champagne | Chardonnay - Veneto | Melon - Loire Valley | Muscat - La Mancha-Alicante | Sauvignon - Loire Valley | Chenin - Loire Valley |
| --- | --- | --- | --- | --- | --- | --- | --- | --- | --- | --- |
| Catechin | 0,8 ± 0,11 ^ab^ | 0,85 ± 0,06 ^ab^ | 0,73 ± 0,119 ^a^ | 0,76 ± 0,046 ^ab^ | 0,56 ± 0,048 ^cd^ | 0,56 ± 0,065 ^cd^ | 0,43 ± 0,015 ^de^ | 0,64 ± 0,052 ^bc^ | 0,37 ± 0,036 ^e^ | 0,35 ± 0,036 ^e^ |
| Epicatechin | 0,73 ± 0,101 ^a^ | 0,72 ± 0,068 ^ab^ | 0,62 ± 0,078 ^a^ | 0,57 ± 0,032 ^bc^ | 0,63 ± 0,031 ^a^ | 0,67 ± 0,051 ^ab^ | 0,56 ± 0,034 ^bc^ | 0,46 ± 0,01 ^cd^ | 0,33 ± 0,024 ^d^ | 0,32 ± 0,036 ^d^ |
| Procyanidin B1 | 0,49 ± 0,02 ^a^ | 0,44 ± 0,025 ^a^ | 0,49 ± 0,05 ^bc^ | 0,47 ± 0,032 ^ab^ | 0,34 ± 0,028 ^cd^ | 0,32 ± 0,014 ^de^ | 0,2 ± 0,012 ^fg^ | 0,22 ± 0,037 ^ef^ | 0,14 ± 0,009 ^gh^ | 0,13 ± 0,015 ^h^ |
| Procyanidin B2 | 0,54 ± 0,017 ^a^ | 0,45 ± 0,03 ^bc^ | 0,41 ± 0,05 ^ab^ | 0,39 ± 0,03 ^cd^ | 0,4 ± 0,03 ^c^ | 0,4 ± 0,028 ^bc^ | 0,31 ± 0,027 ^de^ | 0,22 ± 0,034 ^ef^ | 0,16 ± 0,013 ^f^ | 0,14 ± 0,022 ^f^ |
| Procyanidin B3 | 0,32 ± 0,026 ^a^ | 0,21 ± 0,012 ^ab^ | 0,31 ± 0,038 ^c^ | 0,24 ± 0,016 ^bc^ | 0,14 ± 0,015 ^d^ | 0,1 ± 0,007 ^ef^ | 0,12 ± 0,011 ^de^ | 0,15 ± 0,031 ^d^ | 0,07 ± 0,005 ^fg^ | 0,06 ± 0,01 ^g^ |
| Procyanidin B4 | 0,26 ± 0,009 ^a^ | 0,19 ± 0,009 ^ab^ | 0,23 ± 0,027 ^bc^ | 0,17 ± 0,013 ^c^ | 0,13 ± 0,01 ^d^ | 0,13 ± 0,007 ^de^ | 0,14 ± 0,011 ^d^ | 0,09 ± 0,017 ^ef^ | 0,07 ± 0,005 ^fg^ | 0,06 ± 0,01 ^g^ |
| Procyanidin C a | 0,08 ± 0,006 ^a^ | 0,06 ± 0,004 ^a^ | 0,08 ± 0,008 ^c^ | 0,07 ± 0,004 ^b^ | 0,05 ± 0,004 ^d^ | 0,05 ± 0,003 ^d^ | 0,05 ± 0,004 ^de^ | 0,04 ± 0,006 ^e^ | 0,04 ± 0,004 ^ef^ | 0,04 ± 0,007 ^f^ |
| Procyanidin C b | 0,03 ± 0,001 ^ab^ | 0,03 ± 0,002 ^a^ | 0,03 ± 0,003 ^ab^ | 0,03 ± 0,003 ^a^ | 0,03 ± 0,002 ^a^ | 0,03 ± 0,003 ^a^ | 0,02 ± 0,002 ^bc^ | 0,02 ± 0,001 ^c^ | 0,02 ± 0,001 ^c^ | 0,02 ± 0,002 ^c^ |
| Procyanidin C c | 0,37 ± 0,011 ^a^ | 0,31 ± 0,019 ^ab^ | 0,34 ± 0,038 ^b^ | 0,31 ± 0,023 ^b^ | 0,21 ± 0,015 ^c^ | 0,19 ± 0,009 ^cd^ | 0,15 ± 0,011 ^d^ | 0,16 ± 0,03 ^ab^ | 0,1 ± 0,007 ^e^ | 0,09 ± 0,013 ^e^ |
| Procyanidin C d | 0,08 ± 0,003 ^a^ | 0,07 ± 0,005 ^ab^ | 0,08 ± 0,008 ^bc^ | 0,07 ± 0,006 ^bc^ | 0,06 ± 0,004 ^cd^ | 0,05 ± 0,003 ^de^ | 0,04 ± 0,004 ^ef^ | 0,04 ± 0,003 ^fg^ | 0,03 ± 0,002 ^h^ | 0,03 ± 0,002 ^gh^ |
| Procyanidin C e | 0,1 ± 0,004 ^a^ | 0,09 ± 0,006 ^bc^ | 0,08 ± 0,012 ^ab^ | 0,07 ± 0,007 ^cd^ | 0,08 ± 0,008 ^c^ | 0,1 ± 0,008 ^a^ | 0,05 ± 0,002 ^de^ | 0,04 ± 0,005 ^ef^ | 0,03 ± 0,001 ^f^ | 0,03 ± 0,002 ^f^ |
| Procyanidin C f | 0,13 ± 0,01 ^a^ | 0,12 ± 0,008 ^ab^ | 0,12 ± 0,015 ^ab^ | 0,1 ± 0,017 ^bc^ | 0,11 ± 0,017 ^ab^ | 0,13 ± 0,021 ^a^ | 0,07 ± 0,008 ^cd^ | 0,05 ± 0,006 ^d^ | 0,05 ± 0,004 ^d^ | 0,05 ± 0,004 ^d^ |
| Procyanidin C1 | 0,36 ± 0,013 ^a^ | 0,32 ± 0,014 ^cd^ | 0,26 ± 0,025 ^ab^ | 0,24 ± 0,017 ^de^ | 0,25 ± 0,02 ^de^ | 0,28 ± 0,013 ^bc^ | 0,21 ± 0,02 ^ef^ | 0,12 ± 0,021 ^fg^ | 0,1 ± 0,006 ^g^ | 0,09 ± 0,014 ^g^ |
| Procyanidin C h | 0,1 ± 0,004 ^a^ | 0,08 ± 0,004 ^a^ | 0,08 ± 0,009 ^a^ | 0,06 ± 0,005 ^b^ | 0,06 ± 0,009 ^b^ | 0,06 ± 0,004 ^b^ | 0,06 ± 0,005 ^b^ | 0,04 ± 0,005 ^c^ | 0,03 ± 0,004 ^c^ | 0,04 ± 0,002 ^c^ |
| Total | 4,39 ± 0,084 ^a^ | 3,93 ± 0,125 ^b^ | 3,86 ± 0,273 ^b^ | 3,55 ± 0,148 ^b^ | 3,06 ± 0,15 ^c^ | 3,05 ± 0,102 ^c^ | 2,41 ± 0,107 ^d^ | 2,28 ± 0,192 ^d^ | 1,52 ± 0,049 ^e^ | 1,44 ± 0,097 ^e^ |
